# Fingerprint of Characteristic Saffron Compounds as Novel Standardization of Commercial *Crocus sativus* Extracts

**DOI:** 10.3390/foods12081634

**Published:** 2023-04-13

**Authors:** Adal Mena-García, Diego Herrero-Gutiérrez, María L. Sanz, Marina Díez-Municio, Ana I. Ruiz-Matute

**Affiliations:** 1 Instituto de Química Orgánica General (CSIC), Juan de la Cierva 3, 28006 Madrid, Spain; a.mena@iqog.csic.es (A.M.-G.); mlsanz@iqog.csic.es (M.L.S.); 2 Pharmactive Biotech Products, S.L.U. Faraday 7, 28049 Madrid, Spain; dherrero@pharmactive.eu (D.H.-G.); mdiez@pharmactive.eu (M.D.-M.)

**Keywords:** saffron extract, crocin, liquid chromatography, mass spectrometry, standardization, chromatographic profile, fingerprint

## Abstract

Food supplements based on saffron (*Crocus sativus* L.) dried stigma extracts are widely consumed due to their multiple bioactive properties. Saffron extract (SE) standardization is of crucial importance, as it determines the reproducibility of the product quality and is essential for the evaluation of its bioactive effect and safety. Although SEs are commonly standardized considering their safranal content, the lack of specificity of the official methods may give inaccurate measurements. In addition to the development of more precise methodologies, the evaluation of alternative saffron components, such as crocins and picrocrocin, for standardization purposes would also be of interest. Thus, in this study, qualitative and quantitative information regarding picrocrocin and crocin isomers of different commercial saffron extracts was first obtained by a validated methodology using liquid chromatography (HPLC) coupled to diode array (DAD) and mass spectrometer (MS) detectors. Principal component analysis (PCA) was applied to gain insight into the compositional variability and natural grouping of SE. These studies suggested the potential use of the relative content of crocin isomers and *trans*-/*cis*-crocins and *trans*-4 GG/picrocrocin ratios as novel criteria for SE standardization. Their reproducibility and stability under controlled storage conditions for 36 months was demonstrated in a commercial standardized SE (affron^®^).

## 1. Introduction

Saffron spice, obtained from the *Crocus sativus* L. dried stigmas, is highly appreciated by consumers, not only for its culinary uses but also for its health-promoting properties, such as antitumorigenic, cardioprotective, neuroprotective, anti-inflammatory, antioxidant and antidepressant effects, among others [1,2]. In the COVID-19 pandemic scenario and due to the high stress and prolonged periods of isolation that populations were subjected to, saffron properties related to mood elevation, anti-depressant, relaxation and immunity enhancement gained great interest [3,4,5,6]. Thus, different food supplements based on saffron extracts (SE) have emerged in the market in different types of formulations and formats [6].

**Figure 1:**
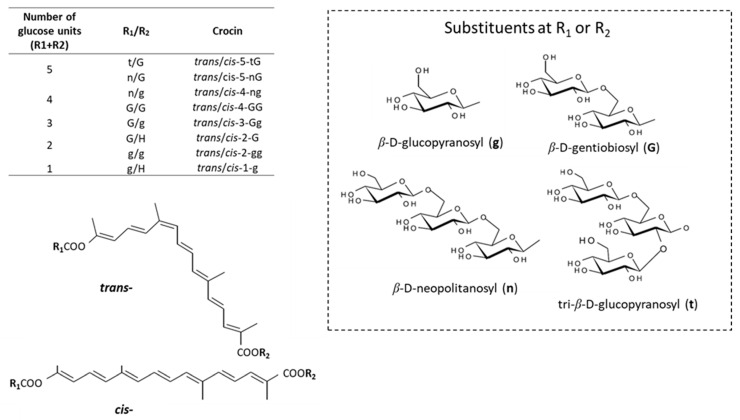
Crocin isomer structures (adapted from Mykhailenko et al. [8]). Nomenclature abbreviations according to Carmona et al. [12].

Most saffron bioactive properties have been associated with its main components: crocins (mono- and bi-esters of crocetin in the *cis*- and *trans*-isomeric forms with different saccharide residues such as glucose, gentiobiose, gentiotriose and neapolitanose, Figure 1), safranal (a monoterpene aldehyde) and picrocrocin (β-d-glucoside of hydroxysafranal) [7,8].

Considering the intrinsic variability of vegetal sources, the standardization of herbal extracts used for the formulation of plant food supplements is essential to preserve the natural phytochemical composition and assure the reproducibility and the quality of the final product [9]. Standardization of herbal extracts is a challenge for the producing companies and involves a complex set of quality controls during all the process, starting from the raw material (botanical specie and plant part used, harvesting and post-harvesting practices, etc.) to the manufacture method, carrying out rigorous controls of the final botanical product to meet a complete set of technical and food safety specifications and to enhance the batch-to-batch reproducibility [10,11]. This is also required for conducting reliable and reproducible clinical trials, where standardized and well characterized materials are desirable in order to allow extrapolating the results to other batches or to similar products [11].

SEs used for the manufacture of commercial food supplements are commonly standardized considering safranal content, and different products of this species are found on the market with a significant variation in it (0.03–2%). However, the official method for safranal quantitation [13], frequently used by producers for the standardization, is based on UV spectrometric determinations, which lacks specificity and can lead to overestimations [14]. Thus, different investigations have been focused on the development of more reliable separative methods such as those based on liquid chromatography (HPLC) coupled to ultraviolet (UV) or mass spectrometric (MS) detectors, not only for quantifying safranal, but also other saffron metabolites such as crocins, picrocrocin, kaempferol and its derivatives [6,15,16,17]. Therefore, an additional approach for saffron extract standardization could be the use of the content of these metabolites. In this sense, some commercial products provide total crocin content as standardization parameter (typically between 3% and 4% (*w*/*w*) of total crocins), although more studies regarding the utility of these markers should be carried out.

Currently, up to 16 crocins have been identified in saffron extracts, including both their *cis*- and *trans*-forms (mono- or di-glycosylated), *trans*-4-GG being the most abundant, followed by *trans*-3-Gg [6,8,15,16]. Although in saffron stigmas the proportion of *trans*-crocins is higher than the *cis*-isomers [7,18], the high temperatures and different energy sources used for stigma dehydration to obtain saffron spice can produce an increase of the *cis*-forms [2,12,18,19]. Moreover, crocins have been noted to degrade over time under certain conditions [15,20] and, therefore, the study of the stability of these bioactive compounds is essential to establish the shelf life of the finished product.

To consider crocins as standardization parameters of saffron products, the presence of these compounds in other different sources must be taken into account. This is the case of *Gardenia jasminoides*, which is one of the main saffron adulterants. Both species mainly differ in their crocin proportions, as gardenia fruit have many of the same crocin isomers as saffron [12]. Although some specific gardenia compounds, such as iridoids [16,21], have been proposed as markers of adulteration, the study of the differences in crocin isomer ratios as a new alternative strategy to detect this type of adulterations should also be explored.

Moreover, picrocrocin, one of the most abundant compounds in saffron and which has been described as an authenticity marker of plants from the genus *Crocus* [2], could also be an interesting compound to be considered for the standardization of SE. However, little attention has been paid to its determination in commercial SEs, and research on this compound has mainly focused on its role as a precursor of safranal and on its thermal degradation kinetics during saffron spice drying [18,22].

Therefore, the aim of this work was to evaluate the possibility of standardizing saffron extracts based on their crocin and picrocrocin contents (individually or as relative proportions), determined by HPLC-DAD, in order to obtain reproducible and stable products. For this purpose, a previous characterization of crocin isomers was required. Moreover, the potential of crocin and picrocrocin profile to determine the quality and authenticity of commercial saffron extracts was evaluated using statistical tools, and its reproducibility and stability was also studied.

## 2. Materials and Methods

### 2.1. Reagents and Standards

Analytical standards of *trans*-4-GG crocin (crocetin bis-(gentiobiosyl) ester), *trans*-3-Gg crocin (crocetin gentiobiosyl glucosyl ester) and picrocrocin were purchased from Phytolab (Vestenbergsgreuth, Germany) while *p*-nitroaniline was obtained from Sigma Aldrich (St. Louis, MO, USA). Acetonitrile and methanol, used for the chromatographic analyses, were of HPLC-MS grade and acquired from Scharlau (Barcelona, Spain). Formic acid was obtained from Sigma Aldrich. The ultrapure water used was distilled and purified using the Milli-Q, Advantage A10 system from Millipore (Bedfore, MA, USA).

### 2.2. Crocins Nomenclature

The nomenclature used in this work for crocetin ester is based on the one previously described by Carmona et al. [12]. The isomeric forms *cis*- and *trans*- are indicated at the beginning, followed by a hyphen and the total number of glucose moieties at both extremes of the crocetin base molecule. After a second hyphen, the structures of lateral glycosides are indicated as (t) triglucoside, (n) neapolitanoside, (G) gentibioside or (g) glucoside. All possible crocin structures are depicted in Figure 1.

### 2.3. Saffron Extracts

Different batches (*n* = 10) of dried commercial powdered extracts of saffron (*Crocus sativus* L.) stigmas (SE1-10), branded as affron^®^ and with Lepticrosalides^®^ as the marker compound, were obtained from the company Pharmactive Biotech Products S.L.U. through a proprietary extraction and manufacturing process registered as AFF^®^ ON Cool-Tech, in its own manufacturing facilities (Madrid, Spain). According to the granted patents (Ref. US2019099464A1 and Ref. ES2573542B1), Lepticrosalides^®^ is the patented totum of bioactive compounds present in affron^®^, with a HPLC safranal content up to 0.35% (dry basis) and a HPLC total crocins content up to 6.00% (dry basis).

Other saffron extracts (SE11-21) of different commercial brands were obtained from specialized trade fairs, commercial contacts, etc. Moreover, for comparative purposes, 6 samples of ground dried saffron stigma (SS1-6) of different proven quality were also included in this study as a reference. These samples were pre-tested according to ISO 3632 and European Pharmacopoeia requirements to confirm their quality. In addition, a *Gardenia jasminoides* extract (GE1) standardized in crocins (40%, *w*/*w*) was also acquired. Some examples of the different samples analyzed are depicted in Figure S1.

Finally, a total of 88 affron^®^ extract samples, from different manufactured batches, were also included in the study in order to evaluate the reproducibility of the crocin profile and the ratios proposed as a proof of their potential for the standardization of commercial saffron extracts. All the samples were packaged in food grade LDPE or PA/PE bags, vacuum sealed and stored at room temperature (<25 °C) and sheltered from light and moisture (<60% RH) until their analysis.

### 2.4. Sample Preparation

Samples (50–90 mg) were dissolved in 20 mL of methanol:water (50:50, *v*/*v*). After vortex agitation, samples were sonicated for 10 min at 40 KHz and 100 W with an ultrasound bath (GT Sonic, VGT-1730QTD model) and vortexed again. Temperature was monitored during sonication, and samples were not allowed to reach temperatures over 35 °C. Samples were then filtered through 0.45 μm syringe filters into amber vials before injection into the HPLC system.

### 2.5. Sample Analysis

#### 2.5.1. Qualitative Analysis

Qualitative analysis was performed on two different HPLC systems:

The first one consisted of an Agilent 1260 Infinity II Prime LC System with an autosampler, a quaternary pump, a thermostatized column compartment and a diode array detector (DAD), coupled to a 6125 single quadrupole (Q) mass detector (Agilent Technologies, Santa Clara, CA, USA) equipped with an electrospray ionization (ESI) source. The analytical separation was carried out in a Phenomenex Kinetex PS C18 (100 × 2.1 mm, 2.6 µm; Phenomenex, Cheshire, UK) at 25 °C, using as mobile phase water with 0.01% of formic acid (solvent A) and acetonitrile with 0.01% of formic acid (solvent B) at a flow rate of 0.5 mL min^−1^. The mobile phase linear gradient used was as follows: 0 min, 5% B; 11 min, 40% B; 16 min, 100% B; 18 min, 100% B, followed by a re-equilibration period of 4 min at initial conditions. The UV/Vis spectra were acquired in the 200–700 nm range, and absorbance at 440 nm and 250 nm were used for the identification of crocins and picrocrocin, respectively. ESI source operated in positive-ion mode, using N_2_ (99.95% purity) as a nebulizing gas at a pressure of 35 psig. The capillary voltage, the drying gas (N_2_) flow and temperature and the fragmentor voltage were set at 3 kV, 12 mL min^−1^, 350 °C and 100 V, respectively. Analyses were carried out in SCAN mode (100–1000 *m/z*). Data acquisition and processing were performed using OpenLAB CDS Software (v.2.19.20, Agilent Technologies).

For HPLC-MS/MS analyses, an Agilent 1200 Series LC system (equipped with a binary pump, an autosampler and a column oven) coupled to a 6520 quadrupole-time of flight (QToF) mass spectrometer (Agilent Technologies) provided with an ESI interface working in positive-ion mode was utilized. Analyses were carried out under the same conditions as in the HPLC-DAD-MS, with the exception of flow rate, which was set at 0.2 mL min^−1^. The electrospray voltage, the drying gas temperature and the fragmentor voltage were set at 4.5 kV, 300 °C and 150 V, respectively. Nitrogen (99.5% purity) was used as the nebulizer (207 kPa) and drying gas (6 L min^−1^), whereas nitrogen of higher purity (99.999%) was used as the collision gas. Tandem mass spectra were obtained by collision-induced dissociation (CID), applying collision energies between 10 and 30 eV to the selected precursor ions. Data acquisition and processing were performed using Agilent Mass Hunter Workstation Rev. B.02.00 software. Mass accuracy below 5 ppm was obtained for elemental composition of target compounds.

#### 2.5.2. Quantitative Analysis

Quantitative analysis was performed on an Agilent 1260 Infinity HPLC, including an autosampler, a quaternary pump, an oven and a DAD detector (Agilent Technologies) following the method developed by Caballero-Ortega et al. [23]. For the analysis, 20 µL of samples were injected into a C18 HPLC column (250 × 4.6 mm, 5 μm) (ZORBAX Eclipse Plus, Agilent Technologies, Product 959990-902) that was kept at a temperature of 22 °C during the run. Mobile phases consisted of water with a 15% of acetonitrile (solvent A) and methanol (solvent B) with a flow rate of 1.0 mL min^−1^ and a linear gradient as follows: 0 min, 10% B, and 60 min, 100% B, followed by a re-equilibration period of 10 min at initial conditions. HP Chemstation software was used for data acquisition. UV/Vis spectra at 440 nm were selected for quantitation of crocins and at 250 nm for picrocrocin; *trans*-4-GG standard was used to quantify all crocins isomers. Due to the limited availability and high price of picrocrocin pure standard, *p*-nitroaniline was used for its determination, as reported in previous studies [24]. Calibration curves with standards at 5 different concentrations (1 ppm–200 ppm) were prepared following the external calibration method.

#### 2.5.3. Method Validation

The validation of the analytical procedure was performed in accordance with the guideline of the International Conference on Harmonization (ICH) of technical requirements for registration of pharmaceuticals for human use [25]. Different parameters were considered for the validation of the method used for the quantitation of crocins isomers and picrocrocin. Precision was measured in terms of repeatability and intermediate precision. Repeatability was determined by analyzing 10 samples of affron^®^ from the same batch within the same day at a concentration of 4 mg mL^−1^ and one sample at 3 different concentration levels (1.75 mg mL^−1^, 3.50 mg mL^−1^ and 5.50 mg mL^−1^) within the same day (*n* = 5). Intermediate precision was evaluated by analyzing 3 samples of affron^®^ from the same batch at a concentration of 4 mg mL^−1^ in the same day and comparing them with another three samples prepared and analyzed on a different day. The linearity of the responses was determined considering five concentrations (*n* = 2) in the 0.93–232.43 µg mL^−1^ range for crocins and in the 18.24–243.26 µg mL^−1^ range for picrocrocin. The goodness of the fitting of the calibration curve was evaluated using its correlation coefficient. The recovery of the method was calculated in quintuplicate spiking a blank sample (consisting of maltodextrins) with a known amount of crocin and picrocrocin standards with a concentration of 21.33% (*w*/*w*) of total crocins and 13.04% (*w*/*w*) of picrocrocin. Recovery was expressed as the percentage of the ratio of the experimental values obtained, divided by the theoretical concentration of the mixture. Limits of detection (*LOD*) and quantitation (*LOQ*) were calculated for crocins as 3.3 σ/S and 10 σ/S, respectively, where σ is the standard deviation of the response and S is the slope of the calibration curve.

### 2.6. Stability of Crocin Profile in Commercial Extracts

Storage assays of affron^®^ samples were carried out over 18 and 36 months in a stability chamber (Osworld Scientific Equipments, OSC G-16 model, 450 L capacity, serial number 3936) under ICH Harmonised Tripartite Guideline for long term controlled temperature and humidity conditions (25 °C ± 2 °C/60% RH ± 5% RH), and samples at different times of storage were taken. All samples were analyzed by HPLC-DAD, as indicated below. Experiments were carried out in duplicate.

### 2.7. Statistical Analysis

Principal Component Analysis (PCA) was carried out using Statistica 7.0 software (StatSoft, Inc., Tulsa, OK, USA).

## 3. Results and Discussion

### 3.1. Crocins and Picrocrocin Identification in Saffron Commercial Extract

**Figure 2:**
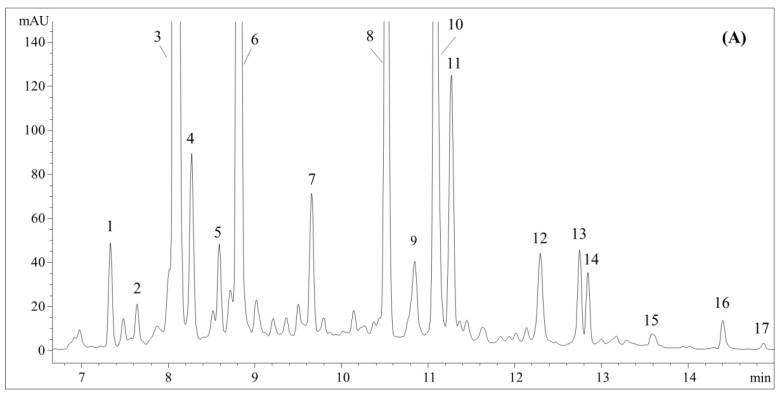

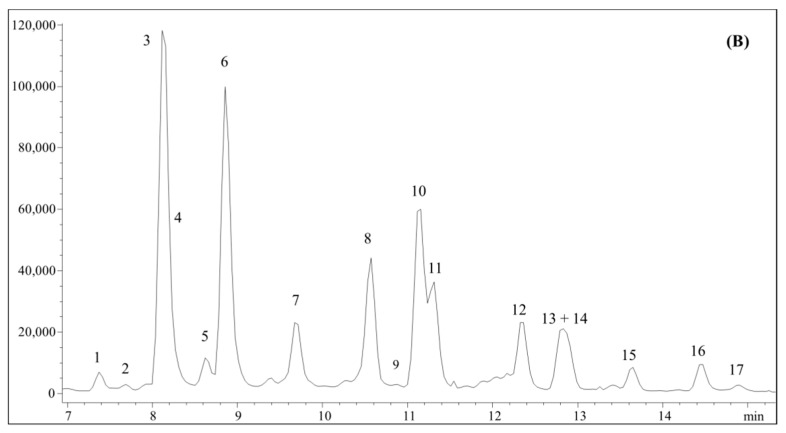
Crocins identified in the standardized saffron extract affron^®^: (**A**) DAD signal at 440 nm; (**B**) MS signal corresponding to the extracted ion chromatogram at 329.1747 *m/z*. The peak assignations can be seen in Table 1.

Although the analysis of crocins and picrocrocin in saffron has been extensively carried out by HPLC-UV [26,27,28,29,30,31] and HPLC-MS [12,32,33,34,35,36], to the extent of our knowledge, very limited information can be found regarding their presence in commercial extracts [6,15,16,17]. Up to 15 crocin isomers were identified in commercial saffron extracts (affron^®^) based on the information obtained by DAD signal at 440 nm (Figure 2A), MS spectra (Figure 2B), accurate mass measurements and the fragmentation pattern obtained by MS/MS analysis. Moreover, the UV/vis spectra allowed the assignation of the *trans*- and *cis*-forms. As previously described by other authors [12,37,38], *trans*-crocins present a main absorption band in their UV/vis spectra at 440 nm, while *cis*-isomers show a hypsochromic effect of 5 nm of their maximum absorption at 440 nm and give an additional absorption band maximum around 325 nm (Figure S2; [39]). Due to the lack of standards of the majority of crocins, these identifications were confirmed based on the relative peak intensities, elution order and fragmentation patterns reported in the literature.

A *m/z* 329.1747 ion corresponding to crocetin [M+H]^+^ species was observed in all crocin product ion spectra (Table 1 and Figure 2B). Moreover, characteristic fragment ions corresponding to different carbohydrate cleavages were also detected.

The elution order observed for crocins in this study followed the pattern described by previous authors for RPLC with a C18 column [12,35]. Low retention times were obtained for more polar crocins with a high number of glucose units, whereas the more folded structure of *trans*-crocin isomers allowed less interaction with the stationary phase and, therefore, these isomers eluted earlier than their respective *cis*-isomers.

The higher molecular weight crocins identified were those with five glucose units, consisting of a gentiobiose (G) and a trisaccharide (a triglucoside (t) or a neapolitanoside (n)) in each crocetin extreme (Figure 1). These assignations were made based on the accurate mass and the fragmentation obtained for peaks 1 and 2 (Figure 2), whose signal at *m/z* ion 1156.4654 was compatible with a molecular formula of [C_50_H_74_O_29_+NH_4_]^+^. Moreover, the fragmentation pattern for both compounds was similar, with *m/z* ions at 832.3602, 653.2805 and 329.1749, corresponding to [M+NH_4_-G]^+^; [M+H-t]^+^ or [M+H-n]^+^ and [M+H-t-G]^+^ or [M+H-n-G]^+^ (Table 1). These peaks were also identified as *trans*-forms based on their UV-visible spectra. Taking also into account data in the literature [12,28,37], peaks 1 and 2 were identified as *trans*-5-tG and *trans*-5-nG, respectively.

Peak 3 was the main signal in the chromatogram, and it was identified as *trans*-4-GG, whose ion at *m/z* 994.4212 corresponds to [C_44_H_64_O_24_+NH_4_]^+^. Moreover, product ions caused by one and two gentiobiose molecule losses were observed (Table 1). Its *cis*-isomer (*cis*-4-GG), labelled as peak 8, showed a similar fragmentation pattern (Table 1). Other crocins with 4 glucose units were detected (peaks 4, 5 and 9) but, instead of giving a loss of disaccharide units, an ion at *m/z* 508.1736 corresponding to a loss of a trisaccharide (triglucoside or neapolitanoside) from one of the crocetin termini was observed. Thus, based on previous studies [28,35], they were assigned as *trans*-triglucosyl glucose crocetin ester (*trans*-4-tg) and *trans*-/*cis*-4-neapolitanosyl glucose crocetin esters (*trans*-/*cis*-4-ng).

Two isomers of crocetin ester with one gentiobiose in one extreme and a glucose in the other (*trans*- and *cis*-3-Gg) were assigned as peaks 6 and 11. Both showed signals corresponding to [C_38_H_54_O_19_+NH_4_]^+^ at *m/z* 832.3598, with fragmentation ions at *m/z* 670.3051 [M+NH_4_-g]+, 329.1753 [M+H-g-G]^+^, 311.1649 [M+H-g-G-H_2_O]^+^ and 293.1538 [M+H-g-G-2H_2_O]^+^, which confirmed their structure.

Regarding crocin structures with two molecules of glucose, two possibilities (both with their respective *cis*-/*trans*-forms) were observed: (i) one glucose at each extreme (2-gg) or (ii) one gentiobiose in one extreme, leaving a free acidic terminus in the other (2-G). In the case of 2-G, its non-glycosylated extreme is affected by the acidic mobile phase used to enhance compound ionization and sensitivity for MS analysis [12,35], and the use of a neutral mobile phase allowed detection of a different chromatographic behavior for this compound, which was useful for the identification of the *trans*- and *cis*-2-G isomers (peaks 10 and 14). Peaks 7 and 12 were identified as *trans*- and *cis*-2-gg, respectively.

Peak 12 was identified as *trans*-1-g based on its sodiated quasi-molecular ion [M+Na]^+^ at *m/z* 513.2112 and the fragments at 329.1753 [M+H-g]^+^, 311.1649 [M+H-g-H_2_O]^+^ and 293.1538 [M+H-g-2H_2_O]^+^. The UV-visible spectrum was used to assign peak 15 as its *cis*-isomer. Finally, the less polar crocetin structures were also detected at the largest retention times and labelled as peaks 16 and 17.

**Figure 3:**
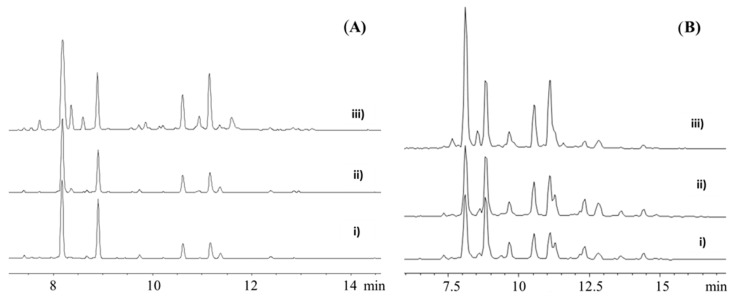
HPLC-DAD-MS analysis of (i) saffron stigmas, (ii) commercial saffron extract (affron^®^) and (iii) gardenia extract; (**A**) DAD signal at 440 nm and (**B**) MS signal corresponding to the extracted ion chromatogram at 329.1747 *m/z*.

Although saffron stigmas are submitted to different processes to obtain the final commercial extracts, including bioactives extraction, blending with excipients and removal of the solvent, as Figure 3 shows, no remarkable qualitative differences were observed in the crocins profile of both saffron stigma (i) and affron^®^ extract (ii), obtained by DAD and MS. This is an important aspect, because these products maintain practically the same full spectrum of crocins as the whole herb, assuring that no major component has been removed in the extraction process and that a minimum level of these bioactive compounds is maintained.

In addition, a gardenia extract standardized in crocins (40%, *w*/*w*) was analyzed, and the profile obtained was compared to that of saffron stigmas and affron^®^ (Figure 3). Although the DAD signal showed the presence of other compounds absorbing at 440nm (Figure 3A), the same major crocins were observed by MS (Figure 3B). Gardenia fruit has been reported as one of the most used saffron adulterants due to its similar crocin profile [16]. However, some quantitative differences in the crocin ratios could be observed.

Picrocrocin was also identified in affron^®^ extract and saffron stigma samples, which showed signals corresponding to [C_16_H_26_O_7_+H]^+^ at *m*/*z* 331.1761 and [C_16_H_26_O_7_+NH_4_]^+^ at *m*/*z* 348.2030, with a fragmentation ion at *m*/*z* 169.1240 [M+H-g]^+^. Identification was confirmed by comparison of the retention time and MS spectrum with that of the pure standard. Picrocrocin was not detected in gardenia extract, as previously reported by Carmona et al. [12].

### 3.2. Crocins and Picrocrocin Quantitation

#### 3.2.1. Validation of the Method

The quantitative determination of crocins and picrocrocin in commercial standardized extracts was carried out by HPLC-DAD after the validation of the method (Table 2). Linear responses were obtained for crocins and picrocrocin within the concentration ranges (0.93–232.43 µg mL^−1^ for crocins and 18.24–243.26 µg mL^−1^ for picrocrocin), with good correlation coefficients (R^2^ = 0.9991 and R^2^ = 0.9998, respectively) for the calibration curves. Regarding the precision of the method, good values for repeatability (RSD of 2.03%/2.14% and 5.54%/1.77%) and intermediate precision (RSD 3.04% and 2.55%) were achieved for both crocins and picrocrocin. Recovery values of 95% for crocins and of 99% for picrocrocin were obtained. *LOD* and *LOQ* values were of 0.13 µg mL^−1^ and 0.40 µg mL^−1^ for crocins and of 3.48 µg mL^−1^ and 10.54 µg mL^−1^ for picrocrocin, respectively. All these parameters indicated the suitability of this method for the quantitation of crocins and picrocrocin in commercial saffron extracts.

#### 3.2.2. Commercial Saffron Extracts

The validated method was then applied for analysis of crocins in different commercial extracts (SE). Moreover, data obtained for different raw saffron stigma samples (SS) and a standardized gardenia extract (GE) were also included for comparison purposes. Table 3 shows the relative content (computed as % of the total content of all crocins) of the seven major crocins found in the samples.

Regarding SE, in all samples, *trans*-crocins were more abundant than their *cis*-isomers. Among them, *trans*-4-GG was the major crocin (36.1–64.7% of total crocins), followed by *trans*-3-Gg (8.6–23.8% of total crocins). Regarding *cis*-isomers, the most abundant compound in all samples was *cis*-4-GG followed by *cis*-3-Gg (3.4–8.8% and 0.8–4.6% of total crocins, respectively). These compounds were also the major crocins determined in stigma samples (SS1–SS6) and agree with that reported in the bibliography [37,40,41]. Regarding gardenia extract (GE1), similar percentages of main crocins were observed; *trans*-4-GG and *trans*-3-Gg were also the most abundant (57% and 10.9% of total crocins, respectively), followed by *trans*-2-G (9.8% of total crocins).

**Figure 4:**
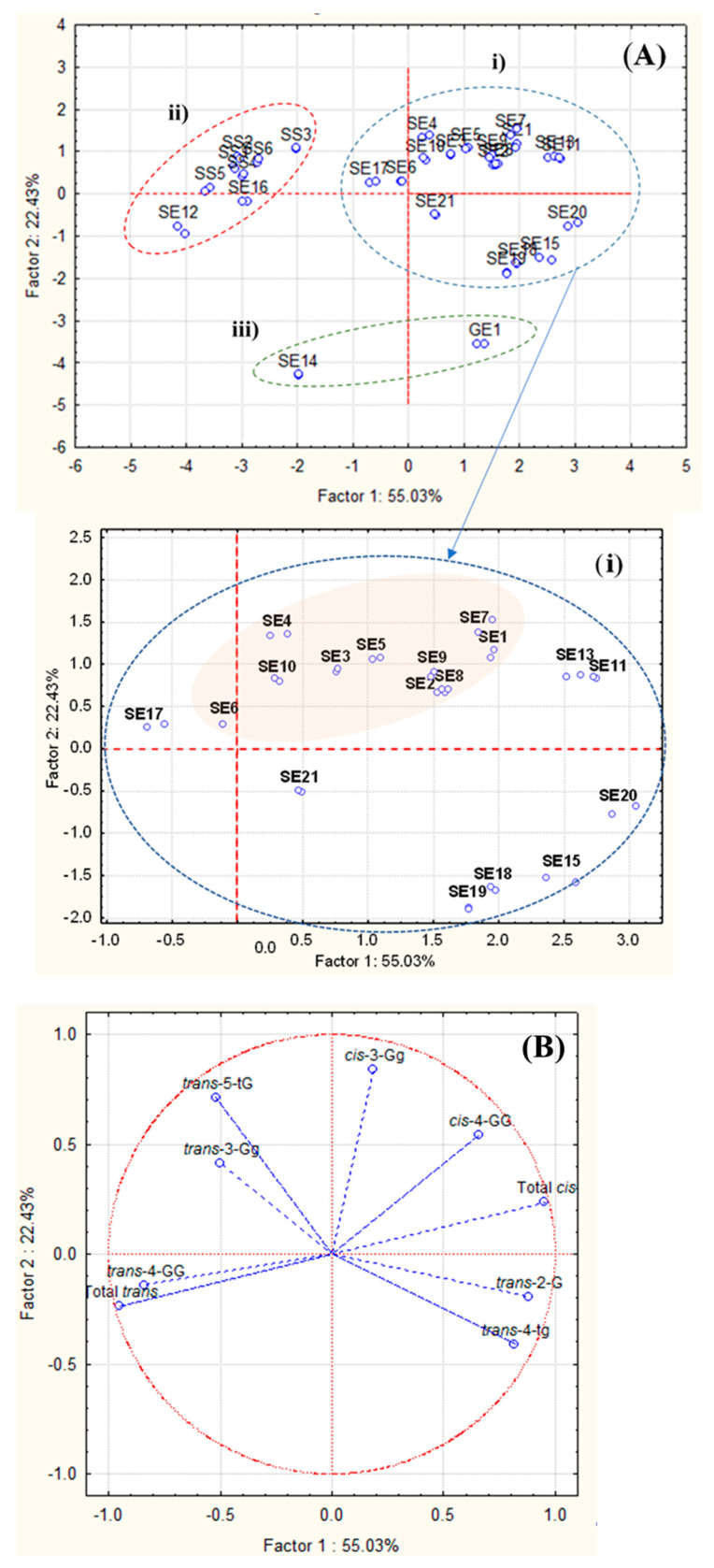
(**A**) Principal component analysis (PCA) biplot of crocin data obtained by HPLC-DAD for commercial saffron extracts (SE), stigmas (SS) and gardenia (GE) under study. (**B**) Projection of the variables on the factor plane (factor 1 *vs.* factor 2).

In order to gain insight into the compositional variability and natural grouping of samples according to their crocin profile, the relative content of the seven major crocins as well as the total *trans*- and *cis*-crocins obtained for the different SE, SS and GE samples were submitted for principal component analysis (PCA). As PC1 *vs.* PC2 plots (Figure 4) show, the first two principal components explained 77.5% of the total variance, which could be reached up to 92.6% considering a third one. The variables that were included for the PCA are shown in Figure 4B, where it can be seen that the ones with more weight in sample segregation were total *trans*-crocins, with a negative score (−0.9534), and total *cis*-crocins, with a positive score (0.9532) on PC1, and *trans*-5-tG and *cis*-3-Gg, having a positive score on PC2 (0.7114 and 0.8406, respectively) (Table S1). Moreover, the high scores of total *trans*-crocins and total *cis*-crocins variables in the component (PC1), which explains most of the variance (55%), indicate their usefulness to explain differences between saffron samples (*trans/cis* ratio values included in Table 3).

The projection of the cases on the factor-plane (Figure 4A) showed that, in general, commercial saffron extracts (i) tended to group separately from saffron stigma samples (ii) and gardenia extract (iii), indicating differences in the content of crocins among the different groups. Commercial saffron extracts were positioned on the top right quadrant of the PCA score plot as a separate group, while stigma and gardenia samples were placed in the top left and in the low right quadrant, respectively. Only three SE samples were differently positioned from the main SE group along the negative side of PC1: SE 12 and SE16, which were close to the cluster of SS samples due to their high *trans/cis* ratio (Table 3), and SE 14, closer to GE in the negative side of PC2, due to its similar *trans*-5-tG and *cis*-3-Gg relative content in addition to its high *trans/cis* ratio. These results could indicate the possible addition to these samples of ground saffron stigma or gardenia extract, respectively. In fact, when considering the absolute content of crocins (Table S2), very high concentrations were detected in SE12 (188.1 mg g^−1^) and SE14 (146.3 mg g^−1^) when comparing to the other commercial extracts (23.8–66.5 mg g^−1^), which were closer to those obtained for the SS (144.3–166.4 mg g^−1^) and GE samples (228.7 mg g^−1^). In contrast, SE16 showed very low concentrations of total crocins (8.2 mg g^−1^).

Considering the important role of picrocrocin in saffron quality and authenticity, this was also determined in the samples under study. As Table 4 shows, no picrocrocin was detected in SE14, confirming the absence of saffron in this sample, which could also indicate a fraudulent use of gardenia instead of saffron as the vegetal source for this extract. In the case of SE12, a higher picrocrocin content (140.4 mg g^−1^) was also observed comparing to the rest of the extracts (20.5–39.7 mg g^−1^) and was also closer to that of SS (89–119.9 mg g^−1^), supporting the hypothesis of a possible addition of ground saffron stigma in this sample. As observed for total crocins, SE16 showed very low amounts of picrocrocin (5.1 mg g^−1^), which, together with the behavior shown by the PCA analysis (Figure 4), could indicate that this extract could be a diluted sample with ground saffron addition.

Taking into account these observations, picrocrocin was also evaluated as a new variable for the PCA analysis (Figure S3, Table 4). In this case, GE and SE were excluded as no picrocrocin was detected in these samples. As shown in the PC1 *vs.* PC2 plot, the first two principal components explained 79.14% of the total variance, where *trans*-4-GG (negative score on PC1 of 0.943) and picrocrocin (positive score on PC2 of 0.838) were the variables having more weight in sample segregation (Table S3). A similar behavior to that previously described in Figure 4 was observed in the new PCA score plot (Figure S3), where saffron stigmas (SS1–SS6), SE12 and SE16 were grouped together on the top left quadrant, while most SE were placed in the right top quadrant. Among them, the samples that showed the lowest *trans*-4 GG/picrocrocin (T4/P) ratios (Table 4), SE13, SE 11 and SE 15, were located furthest to the right in the PC1 *vs.* PC2 plot, while the ones with the higher ratio value were placed in the left zone. Moreover, sample SE21, which had not previously shown considerable differences from other commercial extracts, clearly separated in this PCA (bottom left quadrant) and showed the highest T4/P value (0.96) of the SE samples. The relatively low picrocrocin content in respect of the total crocins of this sample could be due to picrocrocin degradation or even to the addition of an external source of crocins for the enrichment of the extract in these compounds. Thus, *trans*-4-GG/picrocrocin ratio could also be a promising parameter for the discrimination of raw and processed samples and for SE standardization purposes.

### 3.3. Reproducibility and Stability of Picrocrocin and Crocins Chromatographic Profile

In order to evaluate the potential of crocins profile and the proposed *trans/cis* and T4/P ratios as standardization criteria for saffron extracts, they were determined in a total of 88 samples from different manufactured batches of affron^®^ extract. Despite the large number of samples analyzed, reproducible crocin and picrocrocin profiles were obtained (Table S4 in Supplementary Material), demonstrating the robustness of the standardized manufacturing procedures for this product. This can also be observed in the PCA cases plot (close up in Figure 4A and Figure S1), where saffron extracts branded as affron^®^ (SE1-10) are located close to each other in the upper right quadrant. Furthermore, their *trans/cis* ratio and T4/P behaved similarly, with maximum values of 7.26 for *trans/cis* and 0.75 for T4/P and minimum values of 3.91 *trans/cis* and 0.49 for T4/P, demonstrating their reproducibility throughout different production batches.

**Figure 5:**
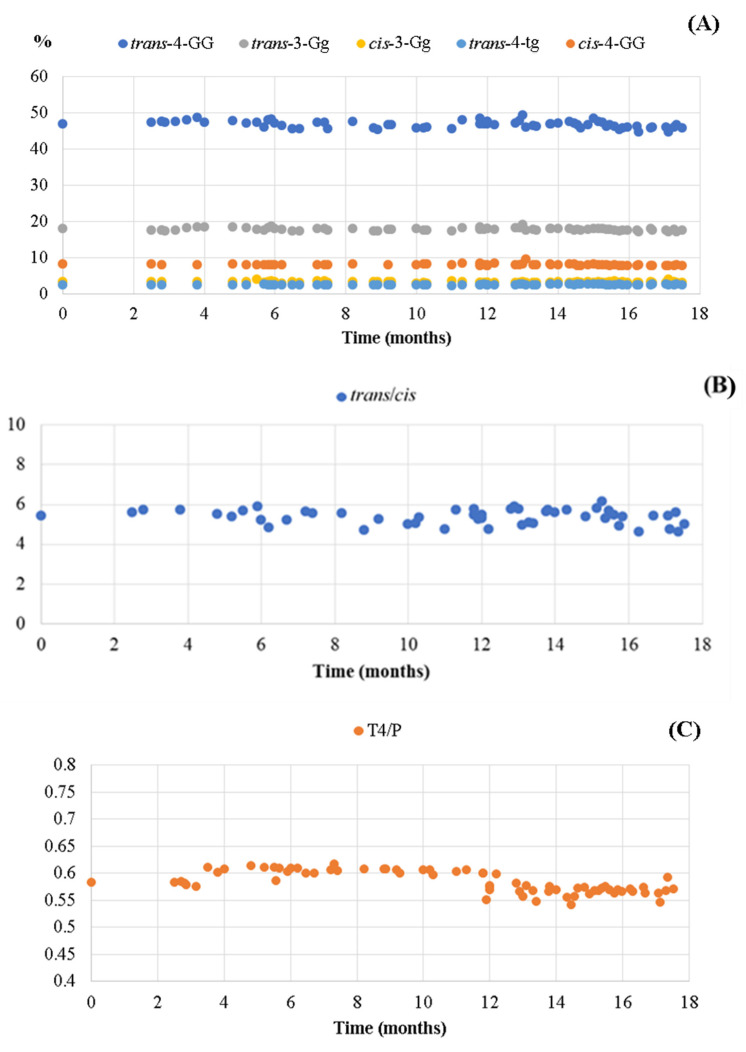
Evolution of crocin isomers, expressed as % of total crocins (**A**); *trans*/*cis* ratio (**B**) and *trans*-4-GG/Picrocrocin (T4/P) ratio (**C**) during storage under controlled conditions (25 °C ± 2 °C, 60% HR ± 5%).

Moreover, as crocins and picrocrocin have been described to be affected by temperature and light exposure during storage [15,42], two affron^®^ samples were stored under controlled temperature and humidity conditions (25 °C ± 2 °C, 60% HR ± 5%) for 18 and 36 months, and their composition was monitored. As can be observed in Figure 5A, practically no variations were observed for the individual crocin isomers (expressed as % of total crocins) determined during the first 18 months. In addition, when initial crocin values where compared to those at 36 months, in general, low variations were observed (RSD < 3.2%). Only *cis*-3Gg showed a higher degradation, its values varying from 3.4% to 2.5% over the 36 months of storage. Similarly, T4/P and *trans/cis* ratios remained stable throughout all the periods evaluated, with RSD < 3.5% and RSD < 6.9%, respectively, in the sample stored for 18 months (Figure 5B,C) and RSD of 15% (T4/P) and of 8.7% (*trans/cis*) for the sample stored for 36 months. This is in accordance with the results reported by Suchareau et al. [15], who described that, contrary to that observed for pure standard, in a saffron extract stored at room temperature, *trans*-4GG was more stable. The presence of other components in these extracts increases the stability and protects crocins from degradation.

## 4. Conclusions

In this study, the standardization of saffron extracts based on their crocin profile and their *trans*/*cis* crocins and *trans*-4-GG/picrocrocin composition, determined by HPLC-DAD, has been demonstrated to be useful for obtaining reproducible and stable products that would allow assuring their quality and evaluating their potential bioactive effect in a reproducible way. Furthermore, the use of the crocins profile and picrocrocin content together with the use of chemometric tools could be a powerful tool to assess the authenticity of saffron extracts. However, although in the particular case of affron^®^ the reproducibility of these parameters throughout different production batches has been very promising, demonstrating the robustness of the standardization process, the content of these markers can vary to some extent depending on the raw material, the extraction method and other processing practices specific for each producer. Therefore, further studies, involving a higher number of samples from different suppliers, and also including a higher number of potential adulterants, is desirable.

## Figures and Tables

**Table 1:** Crocin (Crocetin ester) and crocetin isomers identified in commercial saffron extract affron^®^ by HPLC-Q ToF MS and HPLC-DAD-Q MS.

Nº	Compound	Molecular Formula	Mw	Detected *m*/*z* Ions	Fragmentation (*m/z*)
1	*trans*-5-tG	C_50_H_74_O_29_	1138.4316	1156.4654 [M+NH_4_]^+^	832.3602 [M+NH_4_-G]^+^; 653.2805 [M+H-t]^+^; 329.1749 [M+H-t-G]^+^
2	*trans*-5-nG	C_50_H_74_O_29_	1138.4316	1156.4654 [M+NH_4_]^+^	832.3602 [M+NH_4_-G]^+^; 653.2805 [M+H-n]^+^; 329.1749 [M+H-n-G]^+^
3	*trans*-4-GG	C_44_H_64_O_24_	976.3788	994.4212 [M+NH_4_]^+^	653.2758 [M+H-G]^+^; 635.2687 [M+H-G-H_2_O]^+^; 329.1776 [M+H-2G]^+^; 311.16729 [M+H-2G-H_2_O]^+^; 293.1538 [M+H-2G-2H_2_O]^+^
4	*trans*-4-tg	C_44_H_64_O_24_	976.3788	994.4212 [M+NH_4_]^+^	508.1736 [M+NH_4_-t]^+^; 329.1749 [M+H-t-g]^+^
5	*trans*-4-ng	C_44_H_64_O_24_	976.3788	994.4212 [M+NH_4_]^+^	508.1736 [M+NH_4_-n]^+^; 491.1191 [M+H-n]^+^; 329.1749 [M+H-n-g]^+^
6	*trans*-3-Gg	C_38_H_54_O_19_	814.3259	832.3598 [M+NH_4_]^+^	670.3051 [M+NH_4_-g]^+^; 329.1753 [M+H-g-G]^+^; 311.1649 [M+H-g-G-H_2_O]^+^; 293.1538 [M+H-g-G-2H_2_O]^+^
7	*trans*-2-gg	C_32_H_44_O_14_	652.2731	670.3069 [M+NH_4_]^+^	329.1753 [M+H-gg]^+^; 311.1649 [M+H-gg-H_2_O]^+^; 293.1538 [M+H-gg-2H_2_O]^+^
8	*cis*-4-GG	C_44_H_64_O_24_	976.3788	994.4212 [M+NH_4_]^+^	670.3052 [M+NH_4_-G]^+^; 635.2687 [M+H-G-H_2_O]^+^; 329.1776 [M+H-2G]^+^; 311.16729 [M+H-2G-H_2_O]^+^; 293.1538 [M+H-2G-2H_2_O]^+^
9	*cis*-4-ng	C_44_H_64_O_24_	976.3788	994.4212 [M+NH_4_]^+^	653.2758 [M+H-G]^+^; 635.2687 [M+H-G-H_2_O]^+^; 508.1736 [M+NH_4_-n]^+^; 329.1776 [M+H-2G]^+^; 311.16729 [M+H-2G-H_2_O]^+^; 293.1538 [M+H-2G-2H_2_O]^+^
10	*trans*-2-G	C_32_H_44_O_14_	652.2731	670.3069 [M+NH_4_]^+^	329.1753 [M+H-G]^+^; 311.1649 [M+H-G-H_2_O]^+^; 293.1538 [M+H-G-2H_2_O]^+^
11	*cis*-3-Gg	C_38_H_54_O_19_	814.3259	832.3598 [M+NH_4_]^+^	670.3051 [M+NH4-g]^+^; 329.1753 [M+H-g-G]^+^; 311.1649 [M+H-g-G-H_2_O]^+^; 293.1538 [M+H-g-G-2H_2_O]^+^
12	*cis*-2-gg	C_32_H_44_O_14_	652.2731	670.3069 [M+NH_4_]^+^	329.1753 [M+H-gg]^+^; 311.1649 [M+H-gg-H_2_O]^+^; 293.1538 [M+H-gg-2H_2_O]^+^
13	*trans*-1-g	C_26_H_34_O_9_	490.2203	513.2112 [M+Na]^+^	329.1753 [M+H-g]^+^; 311.1649 [M+H-g-H_2_O]^+^; 293.1538 [M+H-g-2H_2_O]^+^
14	*cis*-2-G	C_32_H_44_O_14_	652.2731	670.3069 [M+NH_4_]^+^	329.1753 [M+H-G]^+^; 311.1649 [M+H-G-H_2_O]^+^; 293.1538 [M+H-G-2H_2_O]^+^
15	*cis*-1-g	C_26_H_34_O_9_	490.2203	513.2112 [M+Na]^+^	329.1753 [M+H-g]^+^; 311.1649 [M+H-g-H_2_O]^+^; 293.1538 [M+H-g-2H_2_O]^+^
16	*trans*-crocetin	C_20_H_24_O_4_	328.1675	329.1741 [M+H]^+^	311.16729 [M+H-H_2_O]^+^; 293.1538 [M+H-2H_2_O]^+^
17	*cis*-crocetin	C_20_H_24_O_4_	328.1675	329.1741 [M+H]^+^	311.16729 [M+H-H_2_O]^+^; 293.1538 [M+H-2H_2_O]^+^

**Table 2:** Analytical parameters for the validation of the LC-DAD method for crocins and picrocrocin determination.

	Crocins	Picrocrocin
Calibration curve	y = 157.9x − 305.9 (R^2^ = 0.9991)	y = 79.44x − 59.91 (R^2^ = 0.9998)
Linear range (µg mL^−1^)	0.93–232.43	18.24–243.26
Repeatability (RSD %)	2.03 ^a^/2.14 ^b^	5.54 ^a^/1.77 ^b^
Intermediate precision (RSD %)	3.04	2.55
*LOD* (µg mL^−1^)	0.13	3.48
*LOQ* (µg mL^−1^)	0.40	10.54
Recovery (%)	95 ± 2	99 ± 2

^a^ Calculated from ten replicates at the same concentration; ^b^ Calculated from five replicates at three different concentrations.

**Table 3:** Crocin content (as % of total crocins) of saffron extracts (SE), raw saffron stigmas (SS) and gardenia extract (GE).

ID	%	*trans*- 5-tG	*trans*- 4-GG	*trans*- 4-tg	*trans*- 3-Gg	*cis*- 4-GG	*cis*- 3-Gg	*trans*- 2-G	Total *trans*-	Total *cis*-	*trans*/*cis*
SE1	Mean	0.8	45.17	3.1	17.1	8.0	4.2	8.5	83.44	16.6	5.0
	RSD	0.3	0.04	0.7	0.5	0.4	2.0	0.9	0.02	0.1	0.1
SE2	Mean	0.9	46.0	2.4	17.6	7.8	2.9	8.8	83.3	16.6	5.0
	RSD	2.5	0.4	1.6	0.4	0.5	2.8	2.4	0.2	0.6	0.5
SE3	Mean	0.9	47.3	2.2	18.1	7.7	3.6	8.9	85.3	14.7	5.8
	RSD	0.5	1.0	2.6	0.7	1.7	3.8	2.4	0.5	2.8	3.3
SE4	Mean	1.0	48.0	1.6	18.5	7.8	3.3	8.0	85.6	14.4	5.9
	RSD	2.2	0.5	0.8	0.2	1.0	5.8	2.9	0.1	0.4	0.4
SE5	Mean	0.9	47.2	1.3	18.4	8.1	3.0	9.3	84.7	15.3	5.5
	RSD	2.1	0.2	0.6	0.3	0.2	1.4	3.7	1.0	5.5	6.5
SE6	Mean	0.9	49.1	2.0	19.5	6.8	2.7	7.8	86.5	13.5	6.4
	RSD	1.1	0.3	1.7	0.8	0.1	2.8	0.5	0.2	1.2	1.4
SE7	Mean	0.8	44.6	2.2	16.9	7.9	4.6	10.1	84.00	16.0	5.3
	RSD	0.5	0.3	0.2	0.8	0.3	5.2	3.9	0.04	0.2	0.2
SE8	Mean	0.87	45.8	2.5	17.4	7.9	2.97	8.1	83.1	16.9	4.9
	RSD	0.03	0.2	0.9	0.5	0.1	0.02	0.4	0.1	0.7	0.8
SE9	Mean	0.9	46.0	1.7	17.6	8.1	3.0	9.0	83.3	16.7	5.0
	RSD	0.2	0.2	1.1	0.3	0.1	0.9	0.6	0.1	0.6	0.7
SE10	Mean	0.9	48.1	1.6	18.8	7.5	2.8	7.5	85.2	14.8	5.8
	RSD	0.4	0.3	0.5	0.8	1.2	0.5	0.5	0.1	0.4	0.4
SE11	Mean	0.8	43.9	2.4	16.9	8.8	3.1	10.1	82.0	18.0	4.5
	RSD	0.6	1.3	2.4	1.3	3.4	0.3	0.3	0.9	4.3	5.2
SE12	Mean	1.1	55.8	0.2	22.94	3.4	1.4	6.1	92.53	7.5	12.4
	RSD	5.0	0.2	2.3	0.04	2.2	0.6	0.8	0.04	0.5	0.6
SE13	Mean	0.9	42.4	2.8	16.14	8.0	3.1	9.7	81.1	18.9	4.3
	RSD	0.1	0.2	0.2	0.04	0.4	2.2	1.4	0.2	0.8	1.0
SE14	Mean	0.4	64.7	1.6	8.6	4.6	0.8	5.5	91.45	8.5	10.7
	RSD	1.9	0.2	2.3	0.1	0.1	0.8	1.1	0.02	0.2	0.2
SE15	Mean	0.8	41.98	5.7	14.9	6.1	2.2	10.6	84.4	15.6	5.4
	RSD	0.6	0.01	0.1	0.5	2.8	1.0	7.7	0.2	0.9	1.1
SE16	Mean	1.0	55.1	0.2	21.6	5.0	2.0	6.5	90.7	9.3	9.8
	RSD	0.6	0.1	14.6	0.1	1.1	0.5	4.2	0.1	1.4	1.6
SE17	Mean	1.0	49.5	1.0	19.5	6.2	2.4	7.9	86.6	13.4	6.5
	RSD	0.6	0.5	2.7	0.1	0.3	1.4	2.2	0.2	1.2	1.4
SE18	Mean	0.8	36.5	3.4	22.0	5.5	0.9	12.6	84.17	15.8	5.3
	RSD	0.0	0.1	0.2	0.6	0.6	0.3	1.1	0.02	0.1	0.1
SE19	Mean	0.7	36.1	3.4	23.8	5.8	0.8	12.9	85.5	14.5	5.9
	RSD	0.5	0.8	1.1	0.6	0.5	0.6	0.1	0.2	1.2	1.4
SE20	Mean	0.7	39.2	3.2	19.1	7.3	1.4	11.4	81.1	18.9	4.3
	RSD	0.6	1.2	6.3	1.1	0.2	1.6	0.1	0.7	3.2	3.9
SE21	Mean	1.0	42.75	2.5	21.7	5.8	1.2	9.4	84.9	15.1	5.6
	RSD	0.3	0.01	1.3	0.7	0.8	1.1	0.9	0.1	0.5	0.5
SS1	Mean	1.1	57.1	0.6	23.2	6.0	2.4	5.7	91.05	9.0	10.2
	RSD	0.9	0.2	1.4	0.4	0.5	0.2	1.2	0.03	0.3	0.3
SS2	Mean	1.2	57.2	0.5	23.1	6.2	2.4	5.7	90.77	9.2	9.8
	RSD	0.8	0.0	1.1	0.1	0.1	0.1	0.5	0.01	0.1	0.1
SS3	Mean	1.0	55.9	0.5	23.0	7.2	2.9	5.8	89.17	10.8	8.2
	RSD	0.2	0.1	0.1	0.2	0.3	0.2	0.5	0.02	0.2	0.2
SS4	Mean	1.0	57.5	0.6	23.4	6.1	2.4	5.5	90.9	9.1	10.0
	RSD	0.8	0.3	2.5	0.3	0.2	0.0	0.3	0.0	0.3	0.3
SS5	Mean	1.0	57.9	0.6	24.7	5.4	2.2	4.8	91.9	8.1	11.4
	RSD	0.2	0.3	1.5	0.2	1.2	1.6	0.3	0.1	1.4	1.5
SS6	Mean	1.0	56.67	0.6	24.1	6.5	2.8	5.2	90.2	9.8	9.2
	RSD	0.4	0.04	1.5	0.1	1.5	0.9	2.7	0.1	1.1	1.2
GE1	Mean	0.4	57.0	4.5	10.9	7.4	0.9	9.8	87.9	12.1	7.3
	RSD	0.8	0.3	0.5	1.0	0.9	1.7	0.6	0.5	3.8	4.3

**Table 4:** Picrocrocin content (mg g^−1^) and *trans*-4 GG/picrocrocin (T4/P) ratio for saffron extracts (SE), raw saffron stigmas (SS) and gardenia extract (GE). ND: non-detected.

ID		Picrocrocin	T4/P
SE1	Mean	35.3	0.57
	RSD	1.05	1.24
SE2	Mean	32.3	0.62
	RSD	0.79	0.6
SE3	Mean	32.1	0.61
	RSD	0.18	1.77
SE4	Mean	33.2	0.67
	RSD	0.34	0.96
SE5	Mean	35.0	0.62
	RSD	0.47	0.74
SE6	Mean	30.7	0.70
	RSD	0.66	0.72
SE7	Mean	35.2	0.57
	RSD	0.22	0.19
SE8	Mean	33.2	0.61
	RSD	0.36	1.03
SE9	Mean	33.8	0.61
	RSD	1.29	1.03
SE10	Mean	33.2	0.65
	RSD	0.05	0.28
SE11	Mean	29.6	0.53
	RSD	1.92	4.11
SE12	Mean	140.4	0.71
	RSD	1.02	2.1
SE13	Mean	25.8	0.39
	RSD	1.39	0.41
SE14	Mean	ND	ND
	RSD	ND	ND
SE15	Mean	26.4	0.54
	RSD	17.01	0.69
SE16	Mean	5.1	0.86
	RSD	1.74	1.76
SE17	Mean	39.7	0.80
	RSD	0.01	0.94
SE18	Mean	20.5	0.67
	RSD	0.77	0.12
SE19	Mean	15.4	0.81
	RSD	2.25	0.64
SE20	Mean	25.8	0.79
	RSD	0.03	0.004
SE21	Mean	23.7	0.96
	RSD	0.16	0.45
SS1	Mean	102.0	0.81
	RSD	0.09	0.56
SS2	Mean	91.6	0.97
	RSD	0.09	0.25
SS3	Mean	89.0	0.97
	RSD	3.23	0.1
SS4	Mean	105.4	0.91
	RSD	2.77	0.86
SS5	Mean	112.5	0.84
	RSD	4.02	1.66
SS6	Mean	119.9	0.73
	RSD	0.09	1.82
GE1	Mean	ND	ND
	RSD	ND	ND

## Data Availability

Data is contained within the article or Supplementary Material.

## Supplementary Materials

The following supporting information can be downloaded at: https://www.mdpi.com/article/10.3390/foods12081634/s1, Figure S1. Picture of some saffron samples analyzed in this study: (i) saffron stigma SS1, (ii) ground saffron stigma SS1, (iii) affron^®^ saffron extract SE1, (iv) saffron commercial extract SE20, (v) saffron commercial extract SE14, (vi) gardenia extract GE1; Figure S2. Typical UV absorption for *trans*-crocins (A) and *cis*-crocins (B); Figure S3. (A) Principal Component Analysis (PCA) biplot of picrocrocin and crocin data obtained by HPLC-DAD for commercial saffron extracts (SE) and stigmas (SS) under study. (B) Projection of the variables on the factor plane (factor 1 *vs.* factor 2). Table S1. Factor loadings for the PCA analysis considering as variables the major crocins and total *cis*- and *trans*-crocins; Table S2. Total crocin concentrations (mg g^−1^) of saffron extracts (SE), raw saffron stigmas (SS) and gardenia extract (GE); Table S3. Factor loadings for the PCA analysis considering as variables the major crocins and picrocrocin; Table S4. Individual crocin isomers content (% of total crocins), *trans/cis* and *trans*-4-GG/Picrocrocin (T4/P) ratios of affron^®^ extract samples from different batches.
