# Supplementary material for: Fingerprint of Characteristic Saffron Compounds as Novel Standardization of Commercial Crocus sativus Extracts
Source: Foods. 2023 Apr 13;12(8):1634. doi: 10.3390/foods12081634 (PMC10137349; doi:10.3390/foods12081634)
Supplement: Supplementary file 1 [file foods-12-01634-s001.zip › foods-2304174-supplementary--final.pdf]

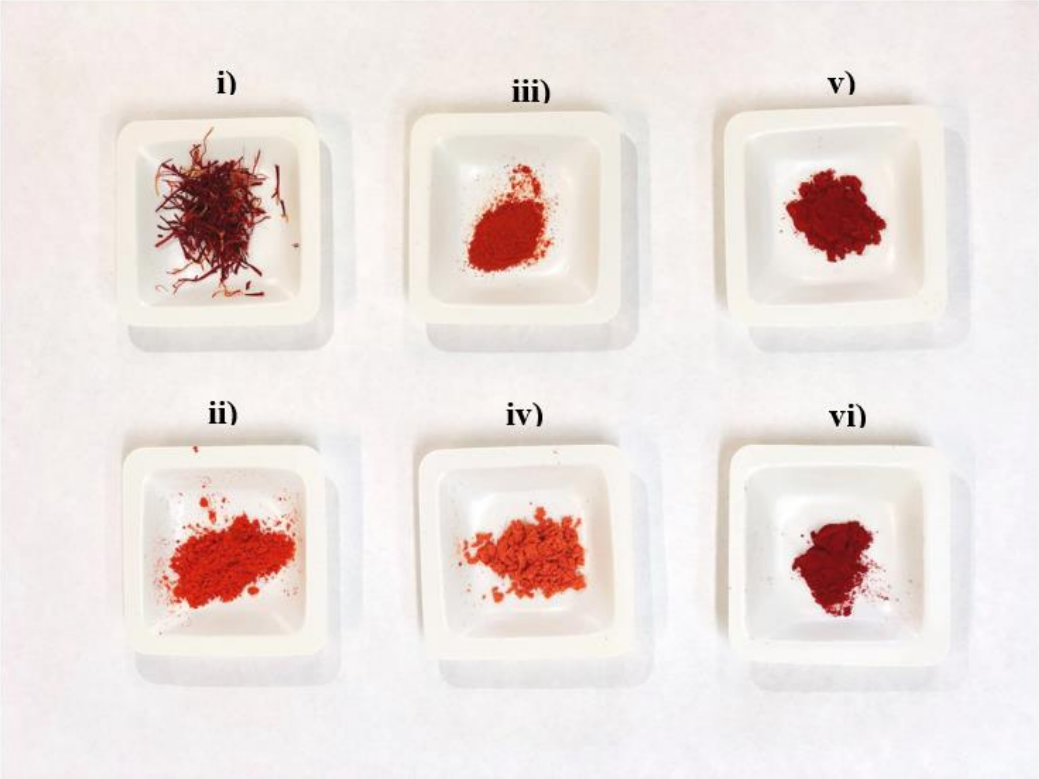

Figure S1. Picture of some saffron samples analyzed in this study: i) saffron stigma SS1, ii) grounded saffron stigma SS1, iii) affron® saffron extract SE1, iv) saffron commercial extract SE20, v) saffron commercial extract SE14, vi) gardenia extract GE1.

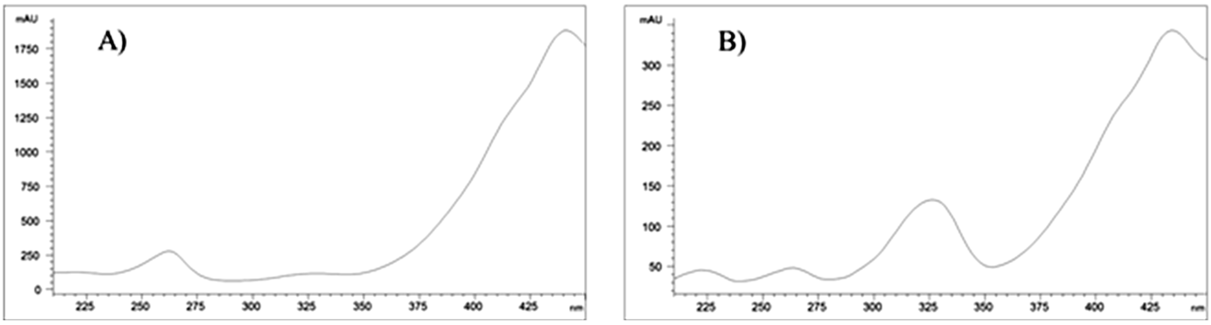

Figure S2. Typical UV absorption for *trans*-crocins (A) and *cis*-crocins (B).

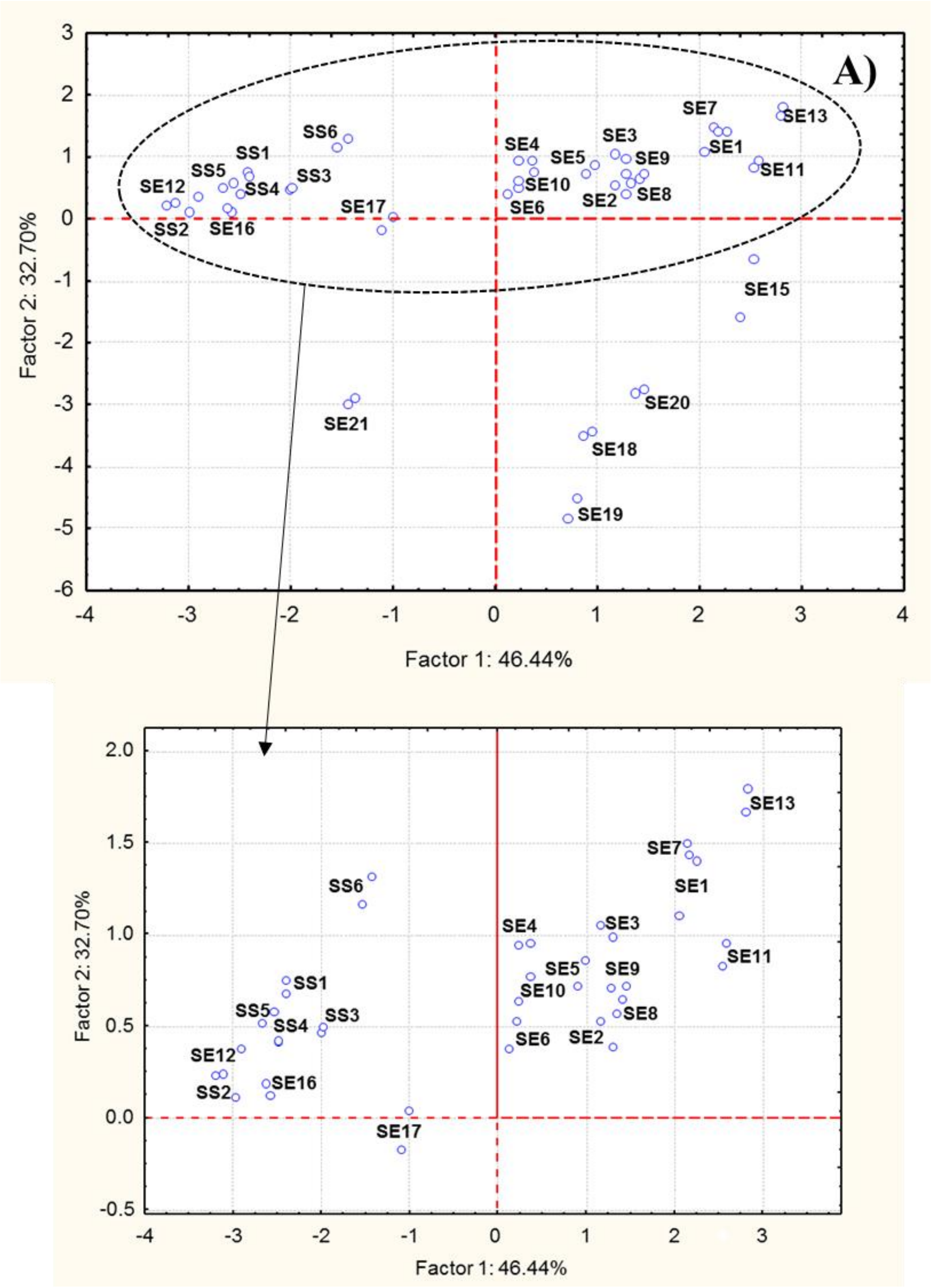
A)
Factor 1: 46.44%
Factor 2: 32.70%
SE1
SE2
SE3
SE4
SE5
SE6
SE7
SE8
SE9
SE10
SE11
SE12
SE13
SE15
SE16
SE17
SE18
SE19
SE20
SE21
SS1
SS2
SS3
SS4
SS5
SS6

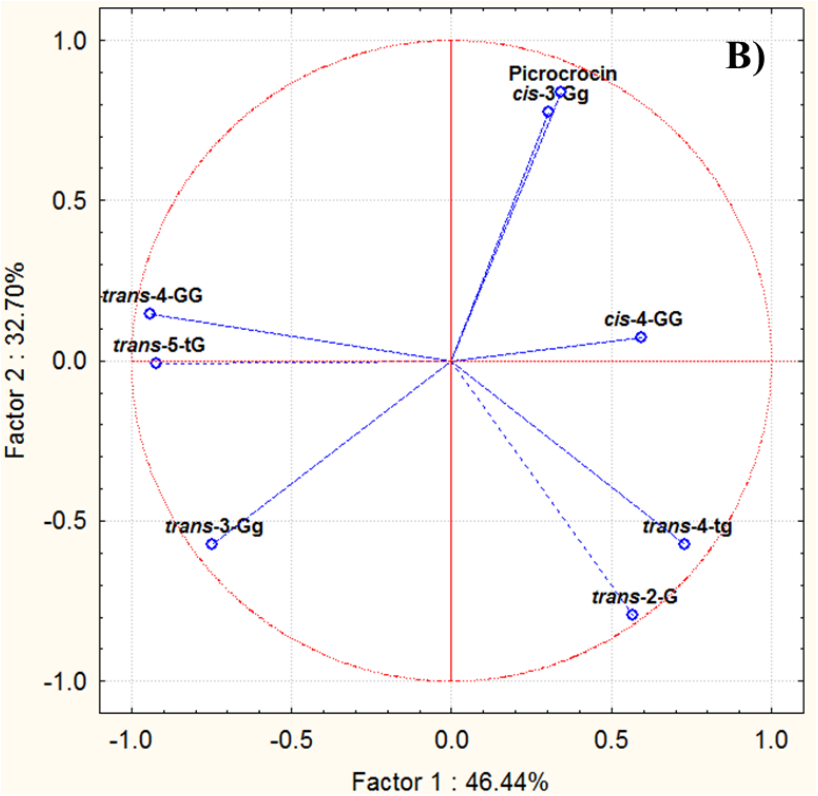

Figure S3. A) Principal Component Analysis (PCA) biplot of picrocrocin and crocin data obtained by HPLC-DAD for commercial saffron extracts (SE) and stigmas (SS) under study. **B)** Projection of the variables on the factor plane (factor 1 *vs* 2).

Table S1. Factor loadings for the PCA analysis considering as variables the major crocins and total *cis*- and *trans*- crocins.

	Factor 1	Factor 2
trans-5-tG	-0.518892	0.711446
trans-4-GG	-0.839354	-0.141232
trans-4-tg	0.817051	-0.410951
trans-3-Gg	-0.502340	0.414796
cis-4-GG	0.659968	0.543370
cis-3-Gg	0.184564	0.840699
trans-2-G	0.878292	-0.193775
Total *trans*	-0.953207	-0.236862
Total *cis*	0.953447	0.236404

Table S2. Total crocin concentrations (mg g^{-1}) of saffron extracts (SE), raw saffron stigmas (SS) and gardenia extract (GE).

ID		Total crocins
SE1	Mean	46.5
	RSD	4.85
SE2	Mean	44.0
	RSD	3.57
SE3	Mean	42.7
	RSD	2.59
SE4	Mean	46.8
	RSD	1.45
SE5	Mean	46.5
	RSD	0.25
SE6	Mean	42.2
	RSD	4.17
SE7	Mean	46.1
	RSD	3.59
SE8	Mean	45.5
	RSD	3.01
SE9	Mean	47.1
	RSD	4.43
SE10	Mean	47.2
	RSD	2.85
SE11	Mean	35.6
	RSD	4.09
SE12	Mean	188.1
	RSD	5.21
SE13	Mean	23.8
	RSD	2.49
SE14	Mean	146.3
	RSD	5.62
SE15	Mean	37.4
	RSD	3.51
SE16	Mean	8.2
	RSD	0.79
SE17	Mean	66.5
	RSD	4.72
SE18	Mean	37.9
	RSD	2.26
SE19	Mean	35.8
	RSD	3.52
SE20	Mean	52.7
	RSD	3.13
SE21	Mean	54.6
	RSD	2.49
SS1	Mean	144.3

	RSD	0.68
SS2	Mean	155.5
	RSD	0.36
SS3	Mean	155.3
	RSD	3.42
SS4	Mean	166.4
	RSD	2.21
SS5	Mean	163.4
	RSD	2.71
SS6	Mean	155.2
	RSD	1.95
GE1	Mean	228.7
	RSD	3.10

Table S3. Factor loadings for the PCA analysis considering as variables the major crocins and picrocrocin.

	Factor 1	Factor 2
trans-5-tG	-0.921072	-0.008428
trans-4-GG	-0.943208	0.145159
trans-4-tg	0.729629	-0.571789
trans-3-Gg	-0.749598	-0.571766
cis-4-GG	0.593166	0.071111
cis-3-Gg	0.305115	0.777226
trans-2-G	0.565910	-0.793205
Picrocrocin	0.342776	0.838191

Table S4. Individual crocin isomers content (% of total crocins), *trans/cis* and *trans*-4-GG/Picrocrocin (T4/P) ratios of affron® extract samples from different batches.

	trans-5-tG	*trans*-4-GG	*trans*-4-tg	*trans*-3-Gg	*cis*-4-GG	*cis*-3-Gg	*trans*-2-G	total *trans*-	total *cis*-	*trans/cis*	T4/P
Mean values, %	0.93	47.76	2.21	18.41	7.66	3.31	8.59	83.79	16.22	5.59	0.60
Maximum	1.03	51.61	3.34	20.50	9.37	4.72	10.15	87.92	20.36	7.26	0.75
Minimum	0.76	43.91	1.07	16.32	5.94	1.90	7.03	79.65	12.08	3.91	0.49
